# Acceptance of Electronic Medical Records and Associated Factors Among Health Care Workers in Northwest Ethiopia: Cross-Sectional Study

**DOI:** 10.2196/72030

**Published:** 2025-12-23

**Authors:** Asmamaw Ketemaw Tsehay, Kholofelo Lorraine Matlhaba

**Affiliations:** 1 College of Medicine and Health Sciences Bahir Dar University Bahir Dar Ethiopia; 2 University of South Africa Pretoria South Africa

**Keywords:** electronic medical records, health care workers, implementation, northwest Ethiopia, perceived ease of use

## Abstract

**Background:**

Although electronic medical records (EMRs) play a vital role in strengthening the health care system by improving efficiency, data management, and patient care, their development in Ethiopia is still in its early stages. Hence, most public health care facilities manage their patient information using paper-based recording, which results in errors, delays, and reduced service quality.

**Objective:**

This study aims to determine the level of acceptance of the EMR system and describe contributing factors.

**Methods:**

A cross-sectional study was conducted at health care facilities in Bahir City, Northwestern Ethiopia. A total of 322 health workers participated in the study, drawn from 5 health facilities that have implemented the EMR system. Descriptive statistics and bivariate and multivariate binary logistic regression were done to determine factors associated with EMR acceptance computed from mediating factors (perceived ease of use and perceived usefulness), and which is more appropriate in early-stage implementation.

**Results:**

Out of the total 322 respondents, 256 (73%) respondents with 95% CI 67.4-78.2 had a good acceptance of using EMRs. In regression analysis, significant predictors including work experience over 10 years (odds ratio [OR] 14.32, 95% CI 4.60-44.58), income dissatisfaction (OR 0.28, 95% CI 0.10-0.82), owning a personal computer (OR 11.08, 95% CI 4.03-30.24), EMR-specific training (OR 4.71, 95% CI 1.52-14.54), basic electronic health management information system/district health information system 2 training (OR 3.06, 95% CI 1.02-9.17), and system usability (OR 38.24, 95% CI 12.26-119.27) were identified.

**Conclusions:**

The study demonstrated a moderate level of EMR acceptance among health care workers, with system usability identified as the strongest predictor. Significant factors influencing EMR acceptance included longer work experience, ownership of a personal computer, and prior EMR or electronic health management information system/district health information system 2 training. Context-specific strategies are needed to enhance system usability, provide targeted digital health training, and improve access to technological resources in order to support broader EMR adoption in health care settings.

## Introduction

The acceptance and effective use of electronic medical records (EMRs) has become a cornerstone of digital health transformation globally, offering the promise of improved care quality, safety, and efficiency [1]. However, there are many aspects that limit the wider adoption of EMRs in sub-Saharan Africa. These include high procurement and maintenance constraints to introduce the EMRs, lack of incentives for implementation, lack of concern to prioritize, electric power shortage, absence of internet, health professionals’ inadequate computer skills, and absence of strong health care facility infrastructure [2].

EMR acceptance is not only a technical issue but also a sociobehavioral one, influenced by system characteristics, user perceptions, and organizational readiness. Studies suggest that both system design and user-related factors such as perceived usefulness (PU), ease of use, and access play critical roles in determining the extent of EMR use [3].

Although most public health care facilities in Ethiopia manually manage data tasks related to their clinical services, there has been a growing demand to adopt the EMR system in health care facilities [4]. Evidence [5] shows that a lack of strategic planning in the field of EMRs had a negative influence on the implementation of the system in health care facilities. Moreover, the Ethiopian Ministry of Health recommended [6] that health care facilities should have a change management strategy that facilitates the smooth transition of the newly introduced EMR system. This strategy will be part of the health care facilities’ strategic paper and shows how the new systems are to be implemented and sustainable for a longer time. There should also be a protocol to select the best performers and to reward those health professionals working on EMRs. The strategy document should also contain principles on data generation and use at the local level for decision-making [6].

And regarding health professionals’ behaviors, PU, and perceived ease of use (PEOU) will influence user acceptance of the EMRs. User acceptance of technology and associated factors has been considered as an important issue that should be presented and understood before introducing and expanding any software applications or systems [7].

The use of only PU and PEOU as measures of technology acceptance is rooted in the Technology Acceptance Model (TAM) developed by Fred Davis [8]. This model proposed that users’ acceptance of new technology is primarily influenced by how useful they perceive it to be in enhancing their job performance (PU) and how easy they believe it is to use (PEOU). In the original TAM, these 2 constructs were found to significantly predict attitudes toward using, which in turn influenced behavioral intention and ultimately actual system use or acceptability of the EMRs.

Motivation and engagement to use EMRs influence the acceptance and attitude of health care workers toward the technology. In turn, the motivation to use the system is influenced by the EMR’s usability, health care workers’ competence, technology skills, confidence, and perceptions. In general, the workplace motivation of health care workers affects work engagement, job satisfaction, and intention to stay in the workplace [9]. The health care workers’ PEOU (eg, accessing patient data) and PU (eg, providing alerts and reminders) are keys to acceptance of EMRs [10]. Since their introduction, EMRs have been a recurrent issue for physicians across all specialties. From user experience to interoperability, nearly every facet of computer-based systems has drawn the ire of those using them. It was found that the amount of time spent in the physician’s inbox and total time using EMRs were associated with higher physician turnover. The American Medical Association found that EMRs contribute to between 11% and 60% of the burnout physicians experienced in 2021 [11]. Since motivation has an important role in the behaviors of health care workers, the adoption, acceptance of, and attitude toward a new technology, such as an EMR system, may be influenced by individual motivation [9].

A study on the Australian EMR system revealed that nurse motivation emerged as the most common factor relating to both perceived barriers and enablers to a new Australian EMR system implementation [9]. The development and implementation of electronic health records (EHRs) involve lots of challenges, including nurses’ stress and negative consequences for patient safety [12]. Designing a user-friendly interface is another challenging task. A poorly designed interface may lead to reduced time efficiency, poor quality of health care delivery, and can become a threat to the patient’s safety as well. Acceptance of EHR by doctors is also perceived as a barrier. This is due to the extra time that doctor spends entering data electronically, which they otherwise can spend treating the patients [13].

The Ethiopian Ministry of Health has played a pivotal role in piloting and scaling up EMR systems across the country. Notably, the University of Gondar Comprehensive Specialized Hospital was selected as a pilot site for EMR deployment, contributing significantly to the development and testing of EMR systems in Ethiopia [14]. One of the EMR systems implemented at the University of Gondar is Bahmni, an open-source EMR platform that aligns with the Ethiopian EHR standards. This initiative has been instrumental in informing the nationwide scale-up of EMR systems, enhancing health care delivery and information management across Ethiopia.

The EMR system in Ethiopia could not go as far as expected due to factors like behavioral and technical [6]. It was also observed that most of the EMR systems in Ethiopia were not interoperable and automated only some aspects of health services. Hence, most public health care facilities in Ethiopia manage their patient information using paper-based recording, which results in errors, delays, and reduced service quality [4]. Therefore, the shift to electronic records is not questionable, for improved standards of care and clinical decision-making [4]. Moreover, some health care workers encountered mistrust of the system. In this regard, there are only a few studies to show EMR acceptance among health care workers in hospitals, and specifically in the contexts of privacy and security concerns [15].

Therefore, this study aims to explore and identify the key factors influencing the acceptance of EMRs among health care workers in Northwest Ethiopia, with the goal of informing strategies to enhance digital health implementation and improve health system performance in the region.

## Methods

### Study Design and Setting

This study used a cross-sectional quantitative design to assess health care workers’ acceptance of EMRs in health care facilities where the system has been implemented. The objective was to systematically evaluate the factors influencing the acceptance and use of EMRs among health care professionals, with a focus on key determinants such as PU, PEOU, and organizational support. The study was conducted in health care facilities located in Bahir Dar city, Northwest Ethiopia. Five health facilities where EMRs had been implemented were included in the study, and the sample was drawn from these sites.

### Study Participants

The study population was all health care workers who are currently working with the EMRs at health care facilities in Bahir Dar City, Northwest Ethiopia. This includes physicians, health officers, nurses, midwives, laboratory professionals, pharmacy professionals, information system officers, and medical data clerks who are working on EMRs. Health care workers who have prior exposure to EMRs for at least 6 months were included in the study.

The sample size was calculated using the finite population correction, considering a total population size of 1848, an assumed proportion (p) of 0.5, a 95% confidence level, and a 5% margin of error (*d*=0.05). Accordingly, the sample size was computed using the statcalc tool in Epi Info (version 7.2.5.0; Centers for Disease Control and Prevention), and the minimum required sample size was determined to be 318. To account for potential nonresponse or dropout, a 5% contingency was added, resulting in a final sample size of n = 318 + (318 × 0.05) = 334.

The sampling method was systematic random sampling. The sampling frame or the master list was a list of 1848 eligible health workers obtained from HR departments of the 5 health care. Each individual was assigned a unique number to create a master list. With a determined sample size of 334, the sampling interval was calculated as K = 1848/334 ≈ 5.5, rounded to 5. A random starting point between 1 and 5 was selected, and every 5th individual from the list was chosen until the sample size was reached, ensuring a systematic and representative selection.

### Data Collection Methods

The data collection was conducted with a self-administered paper-based questionnaire. The questionnaire has 5 sections, of which 2 were Likert-like items based on a scale from “extremely disagree” to “extremely agree.” The other sections are questions regarding information on health care workers’ sociodemographic and EMR implementation-related questions.

Since quantitative data are always numerical in kind or numbers that have a certain intrinsic meaning, the researchers assigned scores to all kinds of variables in the questionnaire. The score number constitutes a shorthand way of expressing a respondent’s answer [16].

The data collection tool was developed by the researcher (Multimedia Appendices 1 and 2), reviewing similar scientific literature on the topic [17-20]. The outcome variable uses a validated tool by Lewis [21], who developed the TAM item formats to measure technology acceptance. A pretest was conducted, and revisions were made accordingly.

EMR acceptance was computed with the 2 main constructs of TAM (PU and PEOU), which are appropriate for this study. Because EMR implementation is in the early stages in the setting, and factors like behavioral intention and social influence may not add significant predictive value, making PU and PEOU sufficient for assessing the EMR acceptance [22]. Combining PU and PEOU into a single index helps to simplify analysis or report general technology acceptance levels, particularly in descriptive studies and when working with limited sample sizes [23]. Moreover, it can help to reduce model complexity while still capturing overall user perception [24].

### Measurements

According to Lewis [21], to provide TAM scores that are consistent with the System Usability Scale and usability metric for user experience metrics, the following formulas were used to put PU and PEOU on a 0-100-point scale, averaging PU and PEOU to get the overall TAM.

- PU = (AVERAGE [TAM01, TAM02, TAM03, TAM04, TAM05, TAM06]–1) (100/6)
- PEOU = (AVERAGE [TAM07, TAM08, TAM09, TAM10, TAM11, TAM12]–1) (100/6)

Accordingly, in this study, perceived usefulness (PU) is computed with the SPSS statistical package as;

- (MEAN [SE01,SE02,SE03,SE04,SE05,SE06]–1) × (100/6) and Perceived ease of use (PEOU) is computed as;
- (MEAN [SE07,SE08,SE09,SE10,SE11,SE12]–1) × (100/6) that converts a 7-point scale to a 100-point scale.

### Data Analysis

As this was a systematic search for meanings, the data were first cleaned and recoded to fit with the model. The data management and analysis were done with the SPSS (version 28.0; IBM Corp) software. Reliability analysis for the internal consistency of items/scales of the outcome variable was done using Cronbach α [25]. Descriptive statistics are applied to analyze the mean, SD, range, and median. The chi-square test was used to identify the relationships between categorical variables. Participant responses were dichotomized into 2 levels with a mean split by the value of the EMR acceptance score, where the variable is split at the mean. Bivariable and multivariable binary logistic regression were done to determine factors associated with EMR acceptance, and a *P* value of .05 was considered statistically significant. Binary logistic regression assumptions were checked before running the model.

As indicated by Zach’s [26] assumptions of the logistic regression, logistic regression is a method that we can use to fit a regression model when the response variable is binary.

One of the assumptions is that the response variable is binary, and in this regard, the logistic regression assumes that the response variable only takes on 2 possible outcomes: low or high acceptance of EMRs. The second assumption was that the observations are independent, and logistic regression assumes that the observations in the dataset are independent of each other. The third assumption was that there was no multicollinearity among explanatory variables, as logistic regression assumes that there is no severe multicollinearity among the explanatory variables. The variance inflation factor indicates whether a predictor has a strong linear relationship with the other predictors. A value of 10 is a good value at which to worry. If the average variance inflation factor is greater than 1, then multicollinearity may be biasing the regression model. The fourth assumption was that there were no extreme outliers.

### Ethical Considerations

Ethics approval for the study was obtained from the College Research Ethics Committee at the College of Human Sciences, University of South Africa, with the National Health Research Ethics Council Registration (Rec-240816-052) and CREC (18033342_CREC_CHS_2023). In addition, permission to access health facilities was granted by the public health institute, Bahir Dar City, Northwest Ethiopia. Moreover, this study ensured the confidentiality, anonymity, privacy, autonomy, justice, beneficence, and nonmaleficence of the study.

## Results

### Sociodemographic Characteristics of the Participants

About 322 participants responded to the questionnaire with a response rate of 96.4% (322/334). The respondents’ age in this study ranges from 23 to 62 years. The mean age of the respondent was 35 years, with an SD of 6.4. Table 1 indicates that 29.5% (95/322) of the respondents were in the age group of 21-30 years, 53.4% (172/322) of the respondents were aged 31-40 years, and 17.1% (55/322) of the respondents were older than 40 years. And, most of the respondents were female, which accounts for 53.4% (172/322).

**Table 1:** Frequency distribution of background characteristics of the respondents (n=322).

Variable and category		Frequency (N=322), n (%)	Mean (SD)
**Age group (years)**			34.75 (6.40)
	21-30	95 (29.5)	
	31-40	172 (53.4)	
	> 40	55 (17.1)	
**Sex**			
	Male	150 (46.6)	—^a^
	Female	172 (53.4)	—
	Total	322 (100.0)	—
**Marital status**			
	Married	204 (63.4)	—
	Single	88 (27.3)	—
	Others	30 (9.3)	—
**Educational level**			
	Diploma and below	76 (23.6)	—
	BSc	196 (60.9)	—
	MSc	50 (15.5)	—
**Work experience as a health professional**			10.8 (5.7)
	Junior (1-2 years)	8 (2.5)	
	Associate (2-5 years)	41 (12.7)	
	Mid-level (5-10 years)	124 (38.5)	
	Senior (above 10 years)	149 (46.3)	
**Experience on EMR^b^**			4.1 (1.8)
	1-5 years	267 (82.9)	
	Above 5 years	55 (17.1)	
	Total	322 (100.0)	
**Health profession**			
	Specialist and above	13 (4.0)	—
	Physician	43 (13.4)	—
	Nurse	161 (50.0)	—
	Pharmacy	23 (7.1)	—
	Laboratory	28 (8.7)	—
	Others	54 (16.8)	—

^a^Not applicable.

^b^EMR: electronic medical record.

The majority of the respondents (204/322, 63.4%) were married, and most of them had an educational level of BSc and above, where 60.9% (196/322) had a BSc Degree, 15.5% (50/322) had an MSc Degree and above (Table 1)*.*

With regard to health professionals, about half of the respondents were nurses, which accounted for 50% (n=161) of the total participants. And, the number of specialists and above was very low, 4% (n=13). Laboratory and pharmacy professionals had nearly similar figures, 8.7% (n=28) and 7.1% (n=23), respectively, as shown in Table 1.

### Information System and EMR Use

More than half of the study participants (213/322, 66.1%) reported owning a personal computer or a laptop. Moreover, this study observed training in basic computer skills among the health care workers and found that about half of the respondents took the training, which is 56.8% (183/322). However, those who frequently use the internet outside the office were 32.9% (106/322), somewhat lower (Table 2)*.*

**Table 2:** Basic computer training and ownership of a computer, and experience with other health information systems among health care workers (n=322).

Variables and category		Frequency (N=322), n (%)
**Own a personal computer**		
	No	108 (33.5)
	Yes	214 (66.5)
**Attended a basic computer training**		
	No	139 (43.2)
	Yes	183 (56.8)
**Attended a training on EMR^a^**		
	No	160 (49.7)
	Yes	162 (50.3)
**EMR training taken**		
	Before starting to work on the EMR	68 (21.1)
	While working on the EMR	94 (29.2)
	No EMR training	160 (49.7)
**Other eHealth trainings**		
	No	240 (74.5)
	Yes	82 (25.5)
**Basic eHMIS^b^ or DHIS2^c^ training**		
	No	182 (56.5)
	Yes	140 (43.5)
**Know about ICD-10^d^**		
	No	265 (82.3)
	Yes	57 (17.7)
**Produce a health service report using EMR**		
	No	193 (59.9)
	Yes	129 (40.1)

^a^EMR: electronic medical record.

^b^eHMIS: electronic health management information system.

^c^DHIS2: district health information system 2.

^d^ICD-10: International Statistical Classification of Diseases, Tenth Revision.

In this regard, about half of the users (160/322, 49.7%) did not receive training on EMR. This will not be a surprise if these providers have a lower EMR acceptance, as most of them believe to learn from their coworkers. This is indicated in Table 2, where most of the health workers (94/322, 29.2%) took the training while working on the EMR system.

Another was training on eHMIS/DHIS2 (electronic health information system/ district health information system 2). DHIS2 is the latest version of eHMIS, mostly practiced by health offices and monitoring and evaluation experts at health facilities to manage health service reports. The study showed that more than half of the respondents (182/322, 56.5%) did not attend the training.

Further advanced knowledge on the EMRs was very limited, as it was indicated by other eHealth trainings and information about *ICD* (*International Classification of Diseases*), a very common terminology standard most EMR vendors are currently using (Table 2).

### EMR Acceptance Among Health Care Workers

EMR acceptance in this study was measured with 12-item variables of PU and PEOU. Each item had a 7-point ordinal scale response options/agreement scale, from extremely disagree on the left to extremely agree on the right. The study was on EMRs implemented in health care facilities; the researcher measured acceptance related to actual use rather than the anticipated (Table 3). Therefore, the level of EMR acceptance for this study was 4.87 (SD 0.83), with the range from 2.92 to 5.75.

**Table 3:** Mean and SD of electronic medical record (EMR) acceptance among health care workers as measured by the 7-point Likert scale.

TAM^a^ and SN^b^			Items to measure EMR acceptance		Mean (SD)
**Perceived usefulness**					
	1	Using Electronic Medical Records in my job enables me to accomplish tasks more quickly.		5.96 (0.748)	
	2	Using Electronic Medical Records improves my job performance.		4.53 (1.038)	
	3	Using Electronic Medical Records in my job increases my productivity.		5.43 (0.694)	
	4	Using Electronic Medical Records enhances my effectiveness on the job.		4.25 (0.877)	
	5	Using Electronic Medical Records makes it easier to do my job.		6.26 (0.597)	
	6	I have found Electronic Medical Records useful in my job.		6.30 (0.780)	
**Perceived ease of use**					
	7	Learning to operate Electronic Medical Records was easy for me.		5.35 (0.985)	
	8	I found it easy to get Electronic Medical Records to do what I want it to do.		4.36 (0.821)	
	9	My interaction with Electronic Medical Records was clear and understandable.		4.16 (0.680)	
	10	I found Electronic Medical Records to be flexible to interact with.		4.00 (0.994)	
	11	It was easy for me to become skillful at using Electronic Medical Records.		3.31 (0.717)	
	12	I found Electronic Medical Records easy to use.		4.56 (1.079)	
**Overall acceptance**					4.87 (0.83)

^a^TAM: Technology Acceptance Model.

^b^SN: serial number.

**Figure 1:**
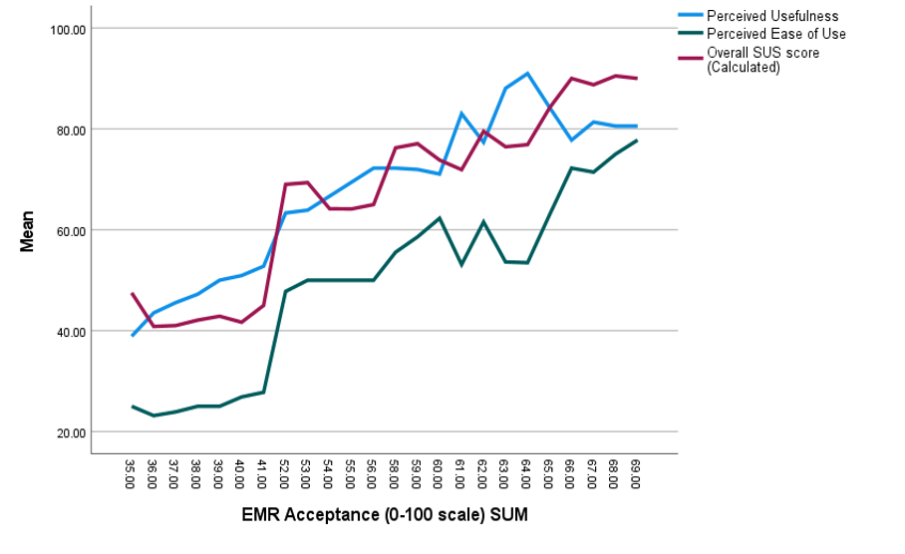
Line graph comparing perceived usefulness, perceived ease of use, and system usability towards EMR acceptance (converted to 0-100 points). EMR: electronic medical record; SUS: System Usability Scale.

When comparing PU with PEOU, the health care workers’ PU score is higher than their PEOU score, as described in Figure 1*.* Among the 6 items from the PU, the item “I have found Electronic Medical Records useful in my job,” has had the highest score of 6.30 from 7.0. Among the items of PEOU, the item, “learning to operate Electronic Medical Records was easy for me,” had the highest score. However, the lowest score (3.31) was about the ease of becoming skillful.

Those health care workers who scored above or equal to the mean score value were categorized as having good EMR acceptance, and those health care workers who scored below the mean value were classified as having poor EMR acceptance.

Out of the total 322 study participants, this study showed that 256 (73%) of the study participants, with a 95% CI of 67.4-78.2, had a good acceptance of using EMRs.

**Figure 2:**
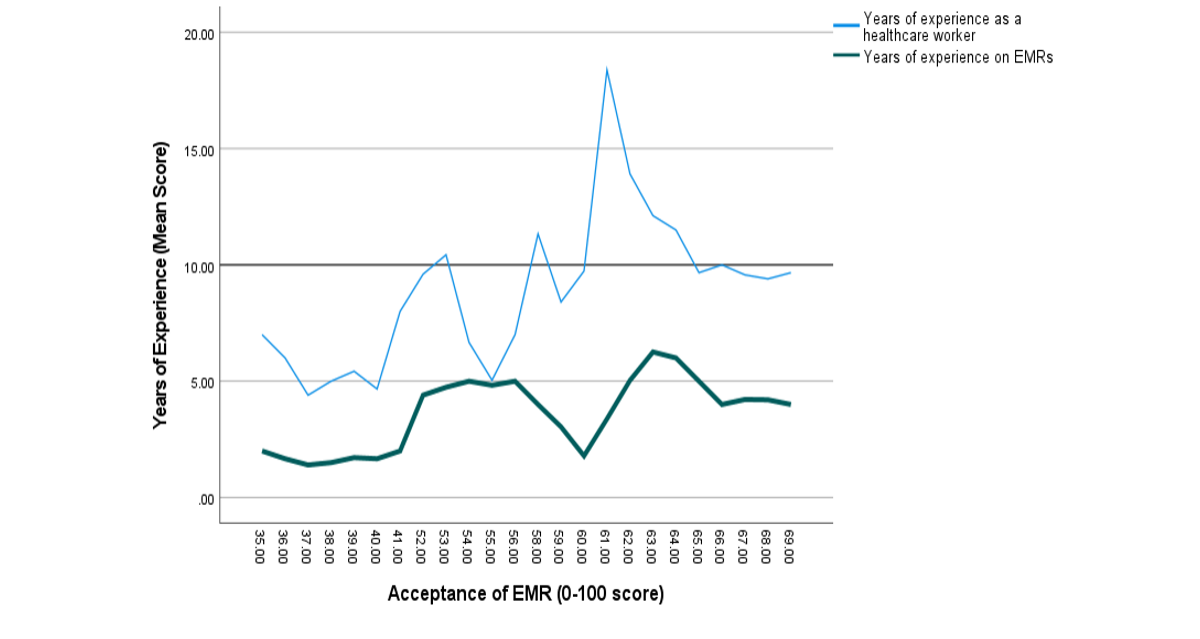
A line graph showing the years of experience as a health care worker versus electronic medical records. EMR: electronic medical record.

Even though the acceptance of EMRs increased with the increase in work experience, as shown in Figure 2, there was a large gap between work experience as a health worker and in EMRs. Lower work experience with EMR is due to the late adoption of the system in the health facilities.

**Figure 3:**
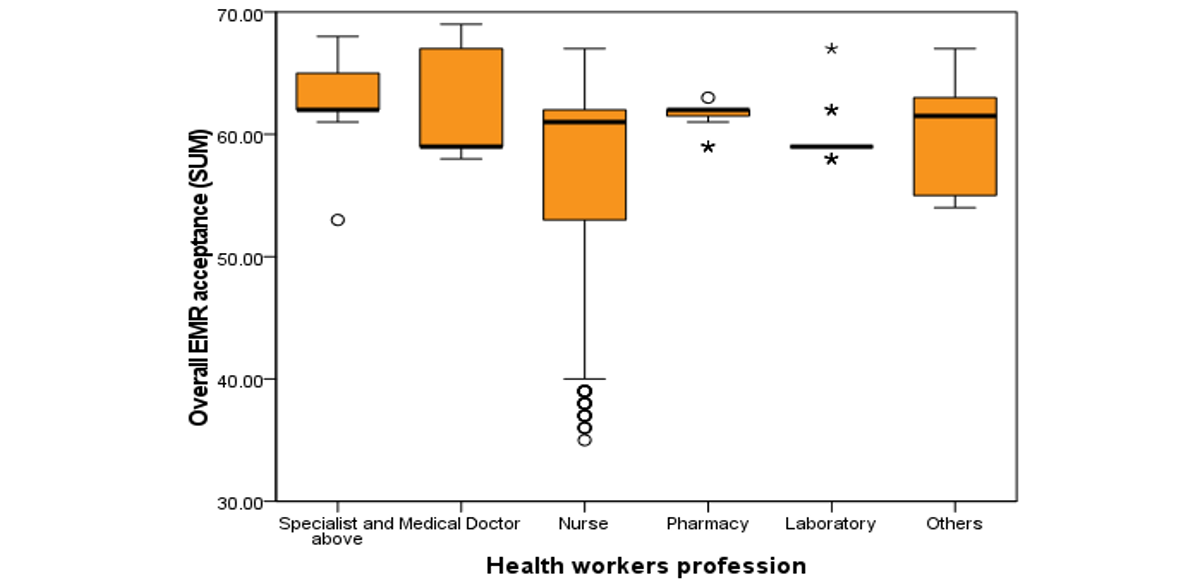
Boxplot showing health professionals’ acceptance of electronic medical records over professional category. “*” indicates an extreme outlier, representing a small number of individuals who have exceptionally divergent views. EMR: electronic medical record.

Higher acceptance of EMRs was observed among pharmacy professionals, specialists, and physicians. This was observed in the boxplot in Figure 3*.*

### Factors Associated With Health Care Workers’ Acceptance of EMRs at Health Care Facilities

To identify predictor variables using a statistical model, it is important to understand the type of dependent (outcome) and explanatory variables. In this study, the dependent variable is the acceptance of EMRs, categorized as either poor or good. Since the researchers’ interest is to examine the factors that determine good acceptance of EMRs, good acceptance is coded as 1 and poor acceptance as 2.

In a binary logistic regression model, the first step is to conduct a univariable analysis to explore the unadjusted association between each independent variable and the outcome. The purposeful selection process begins with a univariate analysis of each variable. Any variable having a significant univariate test at an arbitrary level is selected as a candidate for the multivariable analysis. This selection is based on the Wald test from logistic regression, using a *P* value cutoff point of .25.

As shown in Table 4, both bivariable and multivariable logistic regression analyses were performed to assess the statistical association between the dependent variable (EMR acceptance) and the independent variables. The variables assessed in the bivariable logistic regression included sex, age, years of work experience (grouped), years of experience with EMRs (grouped), ownership of a personal computer, attendance of basic computer training, EMR training, basic eHMIS or DHIS2 training, information about *ICD-10 (International Statistical classification of Diseases, Tenth Revision)*, and EMR system usability (grouped).

**Table 4:** Bivariable and multivariable binary logistic regression to determine factors associated with electronic medical record (EMR) acceptance.

Variables and category			EMR acceptance					COR^a^ (95% CI)			AOR^b^ (95% CI)			*P* value
			Poor		Good									
**Owning a personal computer**														
	No^c^	67		41		1			1			—^d^		
	Yes	20		194		15.85 (8.68 to 28.95)			11.08 (4.03 to 30.42)^e^			<.001		
**Attended a basic computer training**														
	No^c^	58		81		1			1			—		
	Yes	29		154		3.8 (2.26 to 6.40)			0.79 (0.27 to 2.30)			.67		
**Attended a training on EMR**														
	No^c^	60		100		1			1			—		
	Yes	27		135		3 (1.78 to 5.06)			4.71 (1.52 to 14.54)^e^			.007		
**Have basic eHMIS^f^ or DHIS2^g^ training**														
	No^c^	61		121		1			1			—		
	Yes	26		114		2.21 (1.31 to 3.74)			3.06 (1.02 to 9.17)^e^			.046		
**System usability**														
	Low^c^	81		65		1			1			—		
	High	6		170		35.31 (14.69 to 84.88)			38.24 (12.26 to 119.27)^e^			<.001		
**Sex**														
	Male^c^	55		95		1			1			—		
	Female	32		140		2.53 (1.52 to 4.21)			2.68 (0.88 to 8.14)			.08		
**Work experience (years)**														
	1-10^c^	72		107		1			1			—		
	>10	15		128		5.74 (3.11 to 10.60)			14.32 (4.60 to 44.58)^e^			<.001		
**Average monthly income**														
	Unsatisfied^c^	63		135		1			1			—		
	Satisfied	24		100		1.94 (1.14 to 3.33)			0.28 (0.10 to 0.82)			.02		

^a^COR: crude odds ratio.

^b^AOR: adjusted odds ratio.

^c^Reference category.

^d^Not applicable.

^e^Significant at *P*<.05.

^f^eHMIS: electronic health management information system.

^g^DHIS2: district health information system 2.

Variables that were statistically significant at *P*<.25, based on chi-square statistics and the Wald test for individual parameters, were selected for inclusion in the multivariable logistic regression. The Wald statistic helps determine whether a coefficient is significantly different from zero, indicating that the predictor contributes meaningfully to the prediction of the outcome [27,28]. As a result, some variables, namely, age group, experience with EMRs (grouped), and information about *ICD-10*, were excluded from the final model. Finally, to control for potential confounders, a multivariable logistic regression was performed. This was performed by including all relevant confounders in the model; the analysis aimed to reduce bias and provide more accurate and reliable estimates of the associations.

The model test showed that the logistic regression model was statistically significant and able to predict EMR acceptance among health care workers with 73% accuracy (*P*<.001). The model explained between 51.8% (Cox and Snell *R*^2^) and 75.2% (Nagelkerke *R*^2^) of the variance in the confidence score.

Accordingly, the data present results from a multivariable logistic regression analysis examining factors associated with EMR acceptance among health care professionals. The analysis revealed that sex, work experience, access to technology, training, and system usability were key factors influencing EMR acceptance. Although females were more likely to accept EMRs than males in the crude analysis (crude odds ratio 2.53, 95% CI 1.52-4.21), the association was not statistically significant after adjustment (adjusted odds ratio [AOR] 2.68, 95% CI 0.88-8.14, *P*=.08). Health care workers with more than 10 years of experience had significantly higher odds of EMR acceptance compared with those with less experience (AOR 14.32, 95% CI 4.60-44.58, *P*<.001), indicating a strong association.

Owning a personal computer or laptop was a powerful predictor of EMR acceptance. Professionals with personal devices were over 11 times more likely to accept EMRs than those without (AOR 11.08, 95% CI 4.03-30.42, *P*<.001). Similarly, EMR-specific training was significantly associated with higher EMR acceptance (AOR 4.71, 95% CI 1.52-14.54, *P*=.007), as was having basic eHMIS or DHIS2 training (AOR 3.06, 95% CI 1.02-9.17, *P*=.046).

However, attending basic computer training was not significantly associated with EMR acceptance after adjustment (AOR 0.79, 95% CI 0.27-2.30, *P*=.67). The strongest predictor of EMR acceptance was system usability: those who rated the system as highly usable were more than 38 times as likely to accept EMRs compared with those who found it less usable (AOR 38.24, 95% CI 12.26-119.27, *P*<.001).

## Discussion

### Principal Findings

This study aims to examine the factors that affect the acceptance of EMRs among health care workers in Northwest Ethiopia, with the intention of generating evidence to support effective digital health strategies and strengthen health system performance in the region.

In summary, the study found that more than half of the health care workers (256/322, 73%) had a good acceptance of EMR. The factors associated with this were years of work experience, satisfaction with monthly income, owning a personal computer, attending a training on EMR, having basic eHMIS/DHIS2 training, and system usability.

EMR implementation was seen as a challenge for older health professionals, especially those older than 60 years, which may therefore lead to their retention in the workforce [29]. However, in this study, most of the participants were young adults between the ages of 21 and 40 years (172/322, 53.4%), with a SD of 6.4, which is consistent with the study done in Saudi Arabia Hospital, about 62% [30]. Hence, the researcher believed that the acceptance of EMR would be much lower if there were a significant number of older populations found in the sample. Strategies to address technology anxiety, as well as the physical and psychological limitations of an older workforce, are needed to support this workforce during an EMR implementation [29].

More than half of the health care workers (213/322, 66.1%) reported owning a personal computer. However, this was lower than the study done in Oman, where a wide majority of the health care workers who participated in the study, which is 92.3%, reported owning a personal computer or a laptop [31]. Since ownership of technology will improve the PEOU, the health care facility should consider a strategy to enhance technology exposure and support laptops and smartphones for the health care provider.

Not having adequate training and experience is one of the main obstacles to a good EMR implementation and productivity [32]. There was a rapid development of health innovations, where most of them are targeting the point of care. And, sometimes, health professionals may be forced to use the new system without having prior knowledge about it. This can be a factor for low acceptance of EMRs among health care workers. Regarding basic computer training, this study showed that about half of the respondents took the training, (183/322, 56.8%), which is nearly similar to the study in Saudi Arabia hospital, where 52.3% of the participants described that they had formal computer training, while (139/322, 47.7%) mentioned that they did not have any type of computer training [30]. However, the finding is lower than a study done in Muscat, where about 3-quarters of the study participants had computer training. This may affect their ability to competently use the EMR system and influence the quality of interaction with the system while providing health care [31]. It can be explained that having a good experience with information communication technology will help the health care workers to have a higher PEOU, which in turn improves the usability and acceptability of EMRs.

The absence of training and lower experience are among the main obstacles to a good EMR implementation and productivity. In this regard, the study observed EMR training among the health care workers and found that about half of the users did not receive training on EMR (139/322, 49.7%). The magnitude of the problem, the gap in EMR training, will be more visible as one understands how effective the training was. This was described in the Rwandan study, where 98% of respondents agreed that their training on the EHR was effective, and about 93% were confident in using the EMR after having the training [33].

Years of experience as a health professional are another factor that determines EMR acceptance. In this study, about half of the participants (173/322, 54%) had mid and lower-level experience (1-10 years). This is consistent with the study in the Saudi Arabian hospital (56%) [30]. The acceptance of EMR has great paybacks for health professionals, health facilities, and also for the health outcome of the people, if it is used effectively. Therefore, closer attention should be given to the human resource development in line with investing in the introduction of the technology.

EMR acceptance was measured with the latest version of the TAM, 12 items, and a 7-point scale measurement [21]. The level of EMR acceptance for this study was 4.87 (SD 0.83) on a 7-point scale. This finding is lower than a study done in Marie Stops International Clinic in Myanmar among clinical and management staff, with 4.24 (5-point scale) or 5.94 (7-point scale). The scale is converted to compare it with this study [34]. However, it is consistent with the study done in northeastern US regional medical centers among Intensive Care Unit nurses, which was 6.91 (on a 10-point scale), which means 4.83 (on a 7-point scale) [35].

To identify factors associated with the acceptance of the EMR, the variable is recoded and dichotomized into 2 categories (Good and Poor acceptance, with a mean of 4.87, SD 8.3), using cutoff points. Multivariable analysis showed that health care workers with more than 10 years of work experience had a very high effect on EMR acceptance. This finding is supported by much evidence; a report at a government hospital in Muscat stated, health care workers with 6 years and more experience in using the EMR system had 2 times more likely to accept the EMR than their counterparts (odds ratio 2.49, 95% CI 1.23-5.04, *P*=.01). And, another study from Saudi Arabia indicated that notable positive relationship were found between years of experience at the organization and acceptance of EMR [30,31,36].

Owning a personal computer also had a positive effect on acceptance of the EMRs among health care workers. This is consistent with what was done in Belgian Hospital staff, where the length of computer usage was found to have a positive correlation with acceptance of EMR [36]. This may be because health workers’ behavior would be influenced by IT use and being more familiar with computer usage. So, EMR acceptance can sometimes be effective with only a little support to the system users.

Sometimes technology may be introduced into the organization without creating awareness and having training, which leads to hesitancy to use the system. In this study, about half of the participants reported having training on EMR. And, it has a significant effect on accepting EMR, where health care workers who took the training on EMR were 4 times more likely to have a good acceptance than those who did not take the training (AOR 4.71, 95% CI 1.52-14.54; *P*<.007).

### Theoretical and Practical Implications of the Study

The study reinforces the relevance of the TAM in predicting EMR acceptance by health care workers. Key constructs of TAM for this study are PEOU and PU, which are reflected in the factors found to influence EMR acceptance, including prior training, personal computer ownership, and system usability. The findings suggest that individual-level technology experience and training positively shape attitudes toward digital health systems, supporting the theoretical understanding that user readiness and perceived capability are central to successful health information system adoption.

Practically, the study highlights actionable areas for improving EMR implementation in low-resource settings. It emphasizes the importance of investing in digital infrastructure, providing targeted training on EMR systems, and fostering technology exposure among health care workers. Strategies such as supporting staff with personal digital devices, integrating digital health training into ongoing professional development, and addressing the needs of older staff can significantly enhance EMR acceptance. These practical steps can contribute to more efficient health care delivery, improved data quality, and ultimately, better health outcomes.

### Limitations of This Study

This study has some limitations that should be acknowledged. First, the use of a cross-sectional design limits the ability to establish causal relationships between the identified factors and EMR acceptance. While associations can be observed, the temporal sequence of events remains unclear. Future research using longitudinal or experimental designs could better assess causality. Second, the reliance on self-reported data may introduce social desirability and recall biases, which can affect the accuracy of responses, and there may be method bias. Although efforts were made to ensure anonymity and create a nonjudgmental environment to reduce these biases, some degree of misreporting may still have occurred.

### Conclusions

The level of EMR acceptance among health care workers is 4.87 out of a 7-point scale. Though this is above the midpoint, the level of EMR acceptance is somehow lower than other findings.

Higher EMR acceptance was significantly associated with longer work experience, ownership of a personal computer, EMR, or eHMIS/DHIS2 training, and high system usability. Notably, system usability emerged as the strongest predictor. While females showed higher acceptance in the crude analysis, the association was not significant after adjustment. Interestingly, income satisfaction was inversely related to EMR acceptance. These findings highlight the importance of targeted training, access to technology, and user-friendly systems in promoting EMR adoption.

PU and perceived ease determine the level of EMR acceptance. EMR acceptance is the actual use rather than the anticipated use. The health care workers’ PU of the EMR is higher than their PEOU. Years of work experience, being satisfied with monthly income, owning a personal computer, attending training on EMR, having basic eHMIS or DHIS2 training, and system usability were factors that are associated with the acceptance of EMR. It is recommended to have appropriate strategies relevant to the context before implementing the EMRs. Context-specific strategies that enhance system usability, provide targeted training, and improve access to digital tools, particularly for less experienced staff, are essential to increasing EMR acceptance among health care workers.

## Appendix

Multimedia Appendix 1 Questionnaire_English Version.

Multimedia Appendix 2 Questionnaire _ Amharic Version.

## Abbreviations

AOR: adjusted odds ratio
DHIS2: district health information system 2
eHMIS: electronic health management information system
EHR: electronic health record
EMR: electronic medical record
ICD: International Classification of Diseases
ICD-10: International Statistical Classification of Diseases, Tenth Revision
PEOU: perceived ease of use
PU: perceived usefulness
TAM: Technology Acceptance Model

## Data Availability

The data of this study are available from the corresponding author upon reasonable request. Due to privacy, certain data may not be publicly shared. Deidentified data may be shared in accordance with ethical guidelines.
