# Supplementary material for: Acceptance of Electronic Medical Records and Associated Factors Among Health Care Workers in Northwest Ethiopia: Cross-Sectional Study
Source: JMIR Med Inform. 2025 Dec 23;13:e72030. doi: 10.2196/72030 (PMC12775752; doi:10.2196/72030)
Supplement: Multimedia Appendix 1 [file medinform_v13i1e72030_app1.docx]

Questionnaire - English Version

**Title: Electronic Medical Records Acceptance and Implementation among Health Care Workers in Northwest Ethiopia: Developing a scaling up strategy**

I am Asmamaw Ketemaw Tsehay (18033342), and I am doing research with Dr. KL Matlhaba, a professor in the department of health studies towards a PhD in Public Health study in the University of South Africa. Thank you for participating in this study. Kindly complete the questionnaire below.

Instruction for use: Please complete all the sections of the questionnaire.

**Section A: Respondents demographical information**

You are requested to indicate your choice by making a cross (🗶) in the space provided and provide a written answer where there is an open space.

1. I am working in _________________ Health facility
2. I am currently working in _________________ (*specify the department/ ward*)
3. My marital status is ……………

| Single |  |
| --- | --- |
| Married |  |
| Other |  |

1. My current age is _________________ years
2. My professional qualification is……...

| Diploma and below |  |
| --- | --- |
| BSc |  |
| MSc |  |
| PhD and above |  |

1. My profession is _________________ (*specify your profession*)
2. My years of experience as healthcare worker is ___________ (*specify*)
3. My years of experience on EMRs is ___________ (*specify*)
4. My average monthly income is ……...

| Unsatisfactory |  |
| --- | --- |
| Satisfactory |  |

**Section B:Information system and Electronic Medical Records Related Questions**

You are requested to indicate your choice by making a cross (🗶) in the space provided and provide a written answer where there is an open space.

| SN | Question | Response |  |
| --- | --- | --- | --- |
| 1 | I own a personal computer or laptop | No |  |
|  |  | Yes |  |
| 2 | I frequently use internet at office | No |  |
|  |  | Yes |  |
| 3 | I frequently use internet outside the health facility to search important issues related to my work? | No |  |
|  |  | Yes |  |
| 4 | I have EMR training | No |  |
|  |  | Yes |  |
| 5 | If yes to Q. 4, when did you take the training? | Before starting to work on the EMR system |  |
|  |  | While working |  |
|  |  | Other |  |
| 6 | I also take other eHealth trainings | No |  |
|  |  | Yes |  |
| 7 | If yes to Q. 6, Type of training? | Telehealth |  |
|  |  | mHealth |  |
|  |  | Both |  |
|  |  | Others |  |
| 8 | I have fundametal DHIS2/eHMIS training | No |  |
|  |  | Yes |  |
| 9 | I heard about ICD 10 (international classification of disease) | No |  |
|  |  | Yes |  |
| 10 | I have EMR Guideline on my Desk and/or with a soft copy | No |  |
|  |  | Yes |  |
| 11 | I can be able to troubleshoot when there was EMR System failure | No |  |
|  |  | Yes |  |
| 12 | I can generate a health service report with the EMR | No |  |
|  |  | Yes |  |

**Section C: Questions on support and feedback from the health facility**

You are requested to indicate your choice by making a cross (🗶) in the space provided and provide a written answer where there is an open space.

| SN | Question | Response |  |
| --- | --- | --- | --- |
| 1 | Do you believe there was a strong system to monitor the health workers performance on EMR? | No |  |
|  |  | Yes |  |
| 2 | Did you ever get feedback concerning your performance on the EMR? | No |  |
|  |  | Yes |  |
| 3 | Who did you think that takes most of the responsibility to oversee the EMR implementation? | Health Facility |  |
|  |  | District Health Office |  |
|  |  | Regional Health Bureau |  |
|  |  | Federal Ministry of Health |  |
|  |  | NGO |  |
| 4 | Do you think the support is sufficient to the continuity of the EMR? | No |  |
|  |  | Yes |  |
| 5 | Average frequency of supervisory by a monitoring team from the higher level | Weakly |  |
|  |  | Monthly |  |
|  |  | 6Quarterly |  |
|  |  | Biannual |  |
|  |  | Annually |  |
| 6 | Is there EMR performance-based reward in the health facility? | No |  |
|  |  | Yes |  |

**Section D: Electronic Medical Record system usability Questionnaires (SUS)**

The SUS consist of 10 items. You are requested to rate your level of usability according to all the items provided below by making a cross (🗶) in relevant box. The ratings are as follows:1= Strongly Disagree, 2 =Disagree, 3=Neutral, 4= Agree, 5= Strongly Agree.

| SN | SUS Items |  | Ratings:(1= Strongly Disagree, 2 =Disagree, 3=Neutral, 4= Agree, 5= Strongly Agree) | | | |
| --- | --- | --- | --- | --- | --- | --- |
|  |  | 1 | 2 | 3 | 4 | 5 |
| 1 | I think that I would like to use this system frequently |  |  |  |  |  |
| 2 | I found the system unnecessarily complex |  |  |  |  |  |
| 3 | I thought the system was easy to use |  |  |  |  |  |
| 4 | I think that I would need the support of a technical person to be able to use this system |  |  |  |  |  |
| 5 | I found the various functions in this system were well integrated |  |  |  |  |  |
| 6 | I thought there was too much inconsistency in this system |  |  |  |  |  |
| 7 | I would imagine that most people would learn to use this system very quickly |  |  |  |  |  |
| 8 | I found the system very cumbersome to use |  |  |  |  |  |
| 9 | I felt very confident using the system |  |  |  |  |  |
| 10 | I needed to learn a lot of things before I could get going with this system |  |  |  |  |  |

**Section E: Questionnaire/ Electronic Medical Record Acceptance:(Adopted from (Lewis Version 4 TAM).**

(NB: The term /This Product/ in the question is Electronic Medical Record, currently in use by the health workers)

The Technology Acceptance Model (TAM) Scale consist of 12 items. You are requested to rate your experience on EMRs according to all the items provided below by making a cross (🗶) in relevant box. The ratings are as follows:1= Extremely Disagree, 2= Strongly Disagree, 3 =Disagree, 4=Neutral, 5= Agree, 6= Strongly Agree, 7= Extremely Agree.

| SN | TAM Scale Version 4 Items | Ratings:  (1= Extremely Disagree, 2= Strongly Disagree, 3 =Disagree, 4=Neutral, 5= Agree, 6= Strongly Agree, 7= Extremely Agree) | | | | | | |
| --- | --- | --- | --- | --- | --- | --- | --- | --- |
|  |  | 1 | 2 | 3 | 4 | 5 | 6 | 7 |
| Usefulness Items | |  |  |  |  |  |  |  |
| 1 | Using [this product] in my job enables me to accomplish tasks more quickly. |  |  |  |  |  |  |  |
| 2 | Using [this product] improves my job performance. |  |  |  |  |  |  |  |
| 3 | Using [this product] in my job increases my productivity. |  |  |  |  |  |  |  |
| 4 | Using [this product] enhances my effectiveness on the job. |  |  |  |  |  |  |  |
| 5 | Using [this product] makes it easier to do my job. |  |  |  |  |  |  |  |
| 6 | I have found [this product] useful in my job. |  |  |  |  |  |  |  |
| Ease of Use Items | |  |  |  |  |  |  |  |
| 7 | Learning to operate [this product] was easy for me. |  |  |  |  |  |  |  |
| 8 | I found it easy to get [this product] to do what I want it to do. |  |  |  |  |  |  |  |
| 9 | My interaction with [this product] was clear and understandable. |  |  |  |  |  |  |  |
| 10 | I found [this product] to be flexible to interact with. |  |  |  |  |  |  |  |
| 11 | It was easy for me to become skilful at using [this product]. |  |  |  |  |  |  |  |
| 12 | I found [this product] easy to use. |  |  |  |  |  |  |  |
