# Supplementary material for: Acceptance of Electronic Medical Records and Associated Factors Among Health Care Workers in Northwest Ethiopia: Cross-Sectional Study
Source: JMIR Med Inform. 2025 Dec 23;13:e72030. doi: 10.2196/72030 (PMC12775752; doi:10.2196/72030)
Supplement: Multimedia Appendix 2 [file medinform_v13i1e72030_app2.docx]

Questionnaire - Amharic Version

አባሪ ፡ መጠይቅ

ርዕስ ፡ የኤሌክትሮኒክስ የህክምና መዛግብት በኢትዮጵያ ውስጥ በጤና አጠባበቅ ባለሙያ ሠራተኞች ዘንድ ተቀባይነት እና ትግበራ፡ የማሳደጊያ ስትራቴጂን ማዘጋጀት

እኔ አስማማው ከተማው ፀሃይ ነኝ (18033342) እና በደቡብ አፍሪካ ዩኒቨርሲቲ በፐብሊክ ሄልዝ ጥናት ፒኤችዲ በጤና ጥናት ዲፓርትመንት ፕሮፌሰር ከሆኑት ከዶ/ር ኬኤል ማትልሃባ ጋር ጥናት እያደረግሁ ነው ። በዚህ ጥናት ውስጥ ስለተሳተፉ እናመሰግናለን። እባክዎ ከታች ያለውን መጠይቁን ይሙሉ።

የአጠቃቀም መመሪያ፡ እባክዎን ሁሉንም የመጠይቁን ክፍሎች ይሙሉ።

ክፍል ሀ፡ ምላሽ ሰጪዎች የስነ ሕዝብ አወቃቀር መረጃ

በመጠይቁ መሰረት ምልክት በማድረግ ምርጫዎን እንዲጠቁሙ እና ክፍት ቦታ ባለበት የጽሁፍ መልስ እንዲሰጡ ይጠየቃሉ፤

በ ___________________ ጤና ተቋም ውስጥ እየሰራሁ ነው።

___________________ ውስጥ እየሠራሁ ነው (*የስራ ክፍሉን ይግለጹ* )

ጾታ ወንድ ሴት

ዕድሜዬ _____ ዓመት ነው።

የጋብቻ ሁኔታዬ ያገባ. ያላገባ ሌላ

የትምህርት ደረጃ …………

| *ዲፕሎማ እና ከዚያ በታች* |  |
| --- | --- |
| *ቢ.ኤስ.ሲ* |  |
| *ኤም.ኤስ.ሲ* |  |
| *ፒኤችዲ እና ከዚያ በላይ* |  |

የእኔ ሙያ _________________ ነው ( *ሙያዎን ይግለጹ* )

የጤና ባለሙያ ሰራተኛነቴ የዓመታት ልምድ ___________ ነው ( *ይግለጹ* )

EMRs ላይ ያለኝ የዓመታት ልምድ ___________ ነው ( *ይግለጹ* )

አማካይ ወርሃዊ ገቢዬ ___________ ነው፡፡

| *አጥጋቢ ያልሆነ* |  |
| --- | --- |
| *አጥጋቢ* |  |

ክፍል ለ፡ የመረጃ ስርዓት እና የኤሌክትሮኒክስ የህክምና መዝገቦች ተዛማጅ ጥያቄዎች

በመጠይቁ መሰረት ምልክት በማድረግ ምርጫዎን እንዲጠቁሙ እና ክፍት ቦታ ባለበት የጽሁፍ መልስ እንዲሰጡ ይጠየቃሉ፤

| ኤስ.ኤን | ጥያቄ | ምላሽ |  |
| --- | --- | --- | --- |
| 1 | የግል ኮምፒውተር ወይም ላፕቶፕ አለኝ | አይ |  |
|  |  | አዎ |  |
| 2 | ብዙ ጊዜ በይነመረብ በተቋም ውስጥ እጠቀማለሁ | አይ |  |
|  |  | አዎ |  |
| 3 | ከጤና ተቋም ውጭ ብዙ ጊዜ ኢንተርኔት እጠቀማለሁ ከስራ ጋር የተያያዙ ጠቃሚ ጉዳዮችን ፍለጋ | አይ |  |
|  |  | አዎ |  |
| 4 | EMR ላይ ስልጠና ተካፍያለሁ | አይ |  |
|  |  | አዎ |  |
| 5 | ለ Q. 4 አዎ ከሆነ፣ ስልጠናውን መቼ ወሰዱት? | EMR ስርዓት ላይ መስራት ከመጀመሬ በፊት |  |
|  |  | EMR በመስራት ላይ እያለ |  |
|  |  | ሌላ |  |
| 6 | ሌሎች የኢሄልዝ ስልጠናዎችንም ወስደዋል? | አይ |  |
|  |  | አዎ |  |
| 7 | ለ Q. 6 አዎ ከሆነ፣ የስልጠና አይነት? | ቴሌ ሄልዝ |  |
|  |  | ኤም ሄልዝ |  |
|  |  | ሁለቱም |  |
|  |  | ሌሎች |  |
| 8 | eHMIS እና/ወይም DHIS2 ስልጠና ወስደዋል? | አይ |  |
|  |  | አዎ |  |
| 9 | አለማቀፍ የበሽታ ምደባ (ICD 10) ሰምተው ያውቃሉ? | አይ |  |
|  |  | አዎ |  |
| 10 | በእርስዎ ቤሮ እና/ወይም በሶፍት ኮፒ የ EMR መመሪያ አለዎት? | አይ |  |
|  |  | አዎ |  |
| 11 | የ EMR ስርዓት ችግር ሲያጋጥመው መስራት እችላለሁ ብለው ያምናሉ? | አይ |  |
|  |  | አዎ |  |
| 12 | EMR በመጠቀም የጤና አገልግሎት ሪፖርት ማዘጋጀት ይችላሉ? | አይ |  |
|  |  | አዎ |  |

ክፍል ሐ፡- ከጤና ተቋሙ የቀረቡ የድጋፍ እና ክትትል ስራዎችን በተመለከተ ጥያቄዎች እና አስተያየቶች

በመጠይቁ መሰረት ምልክት በማድረግ ምርጫዎን እንዲጠቁሙ እና ክፍት ቦታ ባለበት የጽሁፍ መልስ እንዲሰጡ ይጠየቃሉ፤

| ኤስ.ኤን | ጥያቄ | ምላሽ |  |
| --- | --- | --- | --- |
| 1 | EMR ላይ የጤና ሰራተኞችን አፈፃፀም ለመቆጣጠር ጠንካራ ስርዓት እንዳለ ያምናሉ ? | አይ |  |
|  |  | አዎ |  |
| 2 | EMR ላይ ስላደረጉት አፈጻጸም ግብረመልስ አግኝተው ያውቃሉ ? | አይ |  |
|  |  | አዎ |  |
| 3 | የEMR ትግበራን የመቆጣጠር አብዛኛው ኃላፊነት የሚወስደው ማን ይመስልዎታል ? | የጤና ተቋም |  |
|  |  | የወረዳ ጤና ጥበቃ ቢሮ |  |
|  |  | የክልሉ ጤና ቢሮ |  |
|  |  | የፌዴራል ጤና ጥበቃ ሚኒስቴር |  |
|  |  | መንግሥታዊ ያልሆነ ድርጅት |  |
| 4 | ድጋፉ ለ EMR ቀጣይነት በቂ ነው ብለው ያስባሉ? | አይ |  |
|  |  | አዎ |  |
| 5 | ከበላይ አካላት በክትትል ቡድን አማካኝ የቁጥጥር ድግግሞሽ ምን ይመስላል? | በየሳምንቱ |  |
|  |  | በየወሩ |  |
|  |  | በየሩብ ዓመቱ |  |
|  |  | በዓመት ሁለት ጊዜ |  |
|  |  | በየዓመቱ |  |
| 6 | በጤና ተቋሙ ውስጥ በ EMR አፈጻጸም ላይ የተመሰረተ ሽልማት አለ ? | አይ |  |
|  |  | አዎ |  |

ክፍል D፡ የኤሌክትሮኒክስ የህክምና መዝገብ ስርዓት አጠቃቀም መጠይቆች (SUS)

SUS 10 መለኪያዎችን ያካትታል። በሚመለከተው ሳጥን ውስጥ ምልክት (🗶) በማድረግ ከዚህ በታች በተገለጹት ሁሉም መስፈርቶች መሰረት የአጠቃቀም ደረጃዎን እንዲገመግሙ ተጠይቀዋል ። ደረጃ አሰጣጡ እንደሚከተለው ነው፡ 1= በጣም አልስማማም 2 =አልስማማም 3=ገለልተኛ 4= እስማማለሁ 5= በጣም እስማማለሁ ።

| **ተ.ቁ** | **የ SUS እቃዎች** |  | **ደረጃዎች፡- (1= በጣም አልስማማም፣ 2 =አልስማማም፣ 3=ገለልተኛ፣ 4= እስማማለሁ፣ 5= በጣም እስማማለሁ)** | | | |
| --- | --- | --- | --- | --- | --- | --- |
|  |  | **1** | **2** | **3** | **4** | **5** |
| 1 | ይህንን ስርዓት በተደጋጋሚ መጠቀም እፈልጋለሁ ብዬ አስባለሁ |  |  |  |  |  |
| 2 | ስርዓቱ ሳያስፈልግ ውስብስብ ሆኖ አግኝቼዋለሁ |  |  |  |  |  |
| 3 | ስርዓቱ ለመጠቀም ቀላል እንደሆነ አስብ ነበር |  |  |  |  |  |
| 4 | ይህንን ሥርዓት ለመጠቀም የቴክኒካል ሰው ድጋፍ እንደሚያስፈልገኝ አስባለሁ። |  |  |  |  |  |
| 5 | በዚህ ስርዓት ውስጥ ያሉ የተለያዩ ተግባራት በደንብ የተዋሃዱ ሆነው አግኝቼዋለሁ |  |  |  |  |  |
| 6 | በዚህ ሥርዓት ውስጥ በጣም ብዙ አለመመጣጠን እንዳለ አስቤ ነበር። |  |  |  |  |  |
| 7 | ብዙ ሰዎች ይህንን ስርዓት መጠቀምን ይማራሉ ብዬ አስባለሁ።  በጣም በፍጥነት |  |  |  |  |  |
| 8 | ስርዓቱ ለመጠቀም በጣም አስቸጋሪ ሆኖ አግኝቼዋለሁ |  |  |  |  |  |
| 9 | ስርዓቱን በመጠቀሜ በራስ የመተማመን ስሜት ተሰምቶኛል። |  |  |  |  |  |
| 10 | በዚህ ስርዓት መስራት ከመጀመሬ በፊት ብዙ ነገሮችን መማር ነበረብኝ |  |  |  |  |  |

**የኤሌክትሮኒካዊ የህክምና መዝገብ መቀበል** *፡ (ከ (Lewis Version 4 TAM) የተወሰደ* ።

የቴክኖሎጂ ተቀባይነት ሞዴል (TAM) ልኬት 12 መስፈርቶችን ያካትታል። ከዚህ በታች በተገለጹት ሁሉም መለኪያዎች መሰረት በ EMRs ላይ ያለዎትን ልምድ በምልክት ( 🗶) በሚመለከተው ሳጥን ውስጥ በማድረግ ደረጃ እንዲሰጡ ተጠይቀዋል ። ደረጃ አሰጣጡ እንደሚከተለው ነው፡ 1= እጅግ በጣም አልስማማም 2= በጣም አልስማማም 3=አልስማማም 4=ገለልተኛ 5= እስማማለሁ 6= በጣም እስማማለሁ 7= እጅግ በጣም እስማማለሁ ።

| **ተ.ቁ** | **TAM ልኬት ስሪት 4 መለኪያዎች** | **ደረጃዎች፡- (1= እጅግ በጣም አልስማማም፣ 2= በጣም አልስማማም፣ 3=አልስማማም፣ 4=ገለልተኛ፣ 5= እስማማለሁ፣ 6= በጣም እስማማለሁ፣ 7= እጅግ በጣም እስማማለሁ)** | | | | | | |
| --- | --- | --- | --- | --- | --- | --- | --- | --- |
|  |  | **1** | **2** | **3** | **4** | **5** | **6** | **7** |
| **በጠቃሜታ ዙሪያ ያሉ መለኪያዎች** | |  |  |  |  |  |  |  |
| 1 | በስራዬ ውስጥ [ይህን ምርት] መጠቀም ስራዎችን በፍጥነት እንዳከናውን አስችሎኛል። |  |  |  |  |  |  |  |
| 2 | [ይህን ምርት] መጠቀም የእኔን የስራ አፈጻጸም ያሻሽላል። |  |  |  |  |  |  |  |
| 3 | በስራዬ ውስጥ [ይህን ምርት] መጠቀም ምርታማነቴን ይጨምራል። |  |  |  |  |  |  |  |
| 4 | [ይህን ምርት] መጠቀም በስራው ላይ ውጤታማነቴን ያሳድጋል። |  |  |  |  |  |  |  |
| 5 | [ይህን ምርት] መጠቀም ስራዬን ለመስራት ቀላል ያደርገዋል። |  |  |  |  |  |  |  |
| 6 | በስራዬ ውስጥ [ይህን ምርት] ጠቃሚ ሆኖ አግኝቼዋለሁ። |  |  |  |  |  |  |  |
| **በአጠቃቀም ቀላልነት ዙሪያ ያሉ መለኪያዎች** | |  |  |  |  |  |  |  |
| 7 | [ይህን ምርት] መሥራት መማር ለእኔ ቀላል ነበር። |  |  |  |  |  |  |  |
| 8 | [ይህን ምርት] ማድረግ የምፈልገውን ነገር ለማድረግ ቀላል ሆኖ አግኝቼዋለሁ። |  |  |  |  |  |  |  |
| 9 | ከ [ከዚህ ምርት] ጋር የነበረኝ ግንኙነት ግልጽ እና ለመረዳት የሚቻል ነበር። |  |  |  |  |  |  |  |
| 10 | [ይህ ምርት] ከእሱ ጋር ለመግባባት ተለዋዋጭ ሆኖ አግኝቼዋለሁ። |  |  |  |  |  |  |  |
| 11 | [ይህን ምርት] በመጠቀሜ የተካነ ለመሆን ለእኔ ቀላል ነበር። |  |  |  |  |  |  |  |
| 12 | [ይህን ምርት] ለመጠቀም ቀላል ሆኖ አግኝቼዋለሁ። |  |  |  |  |  |  |  |
